# Association between military service and Alzheimer's disease neuropathology at autopsy

**DOI:** 10.1002/alz.13520

**Published:** 2023-11-15

**Authors:** W. Ryan Powell, Leigha Vilen, Megan Zuelsdorff, Stephen A. Goutman, Shahriar Salamat, Robert A. Rissman, Barbara B. Bendlin, Amy J. H. Kind

**Affiliations:** ^1^ Center for Health Disparities Research University of Wisconsin School of Medicine and Public Health Madison Wisconsin USA; ^2^ Department of Medicine Geriatrics Division University of Wisconsin School of Medicine and Public Health Madison Wisconsin USA; ^3^ Wisconsin Alzheimer's Disease Research Center University of Wisconsin School of Medicine and Public Health Madison Wisconsin USA; ^4^ University of Wisconsin School of Nursing Madison Wisconsin USA; ^5^ Department of Neurology University of Michigan Ann Arbor Michigan USA; ^6^ Department of Neurological Surgery University of Wisconsin School of Medicine and Public Health Madison Wisconsin USA; ^7^ Department of Pathology and Laboratory Medicine University of Wisconsin School of Medicine and Public Health Madison Wisconsin USA; ^8^ Department of Physiology and Neuroscience Alzheimer's Therapeutic Research Institute Keck School of Medicine of the University of Southern California San Diego California USA; ^9^ VA San Diego Healthcare System La Jolla California USA; ^10^ Wisconsin Alzheimer's Institute University of Wisconsin School of Medicine and Public Health Madison Wisconsin USA

**Keywords:** Alzheimer's disease risk, amyloid positivity, military exposome, military risk factors, neurofibrillary pathology, neuropathology, social determinants of health, social exposome

## Abstract

**INTRODUCTION:**

Anti‐amyloid therapies are at the forefront of efforts to treat Alzheimer's disease (AD). Identifying amyloid risk factors may aid screening and intervention strategies. While veterans face increased exposure to risk factors, whether they face a greater neuropathologic amyloid burden is not well understood.

**METHODS:**

Male decedents donating to two Alzheimer's Disease Research Center (ADRC) brain banks from 1986 to 2018 with categorized neuritic plaque density and neurofibrillary tangles (*n* = 597) were included. Using generalized ordered logistic regression we modeled each outcome's association with military history adjusting for age and death year.

**RESULTS:**

Having served in the military (60% of sample) is associated with *post mortem* neuritic amyloid plaque (for each comparison of higher to lower C scores OR = 1.26; 95% confidence interval [CI] = 1.06–1.49) and tau pathology (B score OR = 1.10; 95% CI = 1.08–1.12).

**DISCUSSION:**

This is the first study, to our knowledge, finding increased levels of verified AD neuropathology in those with military service. Targeted veteran AD therapies is a pressing need.

## 1 INTRODUCTION

Military veterans face increased exposures to known Alzheimer's disease and related dementias (ADRD) risk factors. With recent news that the Veterans Health Administration will cover the anti‐amyloid medication Lecanemab, veterans over 65 with amyloid positron emission tomography (PET) or cerebrospinal fluid (CSF) analysis consistent with Alzheimer's disease (AD) and mild cognitive impairment/AD dementia may be eligible to receive biweekly infusions (at an estimated cost of $26,500/year).^1^, ^2^ This landmark decision, coupled with elevated AD risk factors for veterans,^3^, ^4^ ushers in a greater need to describe amyloid burden in veterans relative to non‐veterans as targeted treatments continue to evolve. Barriers exist for such large‐scale surveillance using PET and CSF technologies, but other ways to research and describe amyloid positivity are possible.

Outside of the U.S. Department of Veterans Affairs (VA) and Department of Defense affiliated research, the lack of practical means to connect the military exposome to biobank data is a key obstacle in studying its role in influencing AD biological processes, including amyloid positivity measured at autopsy. This is true of National Institute on Aging (NIA)‐funded Alzheimer's Disease Research Center (ADRC) biobank cohorts, where many ongoing studies are under way in the hopes of making research advancements and new mechanistic discoveries.

### 1.1 The military exposome: A conceptual foundation

The exposome encompasses all exposures external to the individual occurring over the life course that affect health.^5^ Working conditions and contexts over the life course, the occupational exposome, are an important source of exposures and risks.^6^, ^7^, ^8^, ^9^ For those with military service, the military exposome exposes them to a particular set of working conditions and contexts at specific times in their lives, including greater exposure to known and yet to be uncovered risk factors.

The impact of the military exposome on AD risk can be conceptualized and researched in several ways. Military service as a determinant of AD neuropathology may operate broadly and distally, for example, as a marker of chronic stress risk stemming from psychological factors and chronic physical pain.^10^, ^11^, ^12^, ^13^, ^14^, ^15^, ^16^, ^17^, ^18^, ^19^, ^20^, ^21^, ^22^ It may also operate more proximally and specifically as determinants tied to conditions and contexts that have lasting consequences, for example, when there is exposure to physical trauma (eg, traumatic brain injuries) and environmental hazards (Agent Orange).^10^, ^14^, ^16^, ^22^, ^23^, ^24^ The military exposome may also expose individuals to a unique set of heightened exposures to AD risks after service (eg, cardiovascular risk factors).^25^, ^26^

While ADRC cohorts are not universally socially or environmentally categorized in such ways, novel opportunities exist to make such data linkages more feasible. The ability to conduct multilevel analyses^27^, ^28^ of the social‐environmental‐biological interrelationships involving the military exposome and AD risks may result in more effective risk identification and treatment policies.

### 1.2 “New” historic military data via public data tracing

Recent National Institutes of Health‐VA joint efforts to recruit veterans into ADRC studies have opened up opportunities for prospective data collection, but this will take many years to fully realize.^29^ Identifying veterans who participated in brain donation over the last several decades and who already have detailed characterization of their brain tissue at autopsy by a neuropathologist could also hold much promise if military service could be retrospectively identified within a brain donor's life course.

The addition of life‐course risks to existing neuropathological tissue may be possible through public data tracing. Using publicly available sources, research teams can systematically extract historical information from archival records, identifying individuals and potential risk factors stemming from their military service. Demographic data (eg, donor name, birth date, and death date) serve as the starting point for identifying public records. Records include sources commonly used by historians and genealogists to accurately capture military service information according to genealogy standard procedures (eg, military enlistment and pension records, muster rolls, obituaries, newspapers).^30^

Public data‐tracing methods are flexible, allowing researchers to conceptualize and quantify military service in several ways – connecting neuropathology to service‐related conditions, timeframes, activities, and contexts. If more widely available, such novel data linkages can catalyze military exposome research across ADRCs by adding military exposures to longstanding national brain bank repositories.

In this article, we take the initial steps to categorize the military exposome for two ADRC brain banks by identifying those with a military service history via public data tracing and evaluate the association of military service with AD‐related neuropathology, including the presence of amyloid in the brain.

## 2 METHODS

### 2.1 Sample

A cross‐sectional design was used to study 597 male decedents donating their brains to one of two ADRC brain bank programs between 1986 and 2018. Female decedents were included in a sensitivity analysis. University of Wisconsin and University of California San Diego institutional review boards determined the study did not involve human participant research. Data use agreements ensured compliance with protected health information under Health Insurance Portability and Accountability Act of 1996 (HIPAA) regulations. STrengthening the Reporting of OBservational studies in Epidemiology (STROBE) reporting guidelines were followed.

### 2.2 Military service history

Military service history was defined as publicly recorded evidence of any service in the U.S. Armed Forces (binary indicator). Public records were identified through systematic searches of online commercial genealogical databases (Ancestry.com, Newspapers.com, and Fold3.com) and paper archives. Methods are based on genealogical research and unstructured medical record abstraction standards using the Standard for Genealogical Proof.^30^, ^31^, ^32^ Two trained genealogical archivists conducted public data tracing in three phases: (1) independent abstraction by the first archivist, (2) verification of the identity and content of each record and additional independent search by the second archivist, and (3) joint resolution on disagreements. All decedents received this abstraction and validation approach. Some data sources were only available after a fixed window (eg, census 72 years after, military records 62 after last date in service), whereas other sources were available throughout the decedent's lifetime (eg, obituaries, burial records, newspapers articles). Data were extracted between October 2018 and August 2020.

### 2.3 Alzheimer's disease neuropathology

Primary analysis evaluated neurofibrillary tangle and neuritic amyloid plaque pathology. The neuritic amyloid plaque C score and the neurofibrillary tangle B score were either obtained from the standardized neuropathological evaluations using the National Alzheimer's Coordinating Center (NACC) Neuropathology Data Form or abstracted from autopsy reports (primarily for earlier donations prior to the introduction of NACC Neuropathology Data protocols).^33^ Autopsy report abstractions were conducted by two independent, trained abstractors (excellent interrater reliability for Braak Stage before being grouped into B score, Cohen's kappa = 0.99, and amyloid C score = 0.98) with discrepancies resolved by team consensus according to established methods.^31^, ^34^

Using staging per NIA‐Alzheimer's Association (AA) Guidelines, the C score classifies neuritic amyloid plaque presence into four categories (CERAD method): no plaques, sparse, moderate, or frequent.^35^ B scores categorize the deposition of tangles within certain brain regions into four stages (Braak method): B0 (neurofibrillary tangles not present), B1 (Braak Stage I/II; transentorhinal stages), B2 (Braak Stage III/IV; limbic stages), and B3 (Braak Stage V/VI; isocortical stages).^36^ Derived from AD Braak staging using the same guidelines for neuropathologic change,^35^ B scores were chosen because they have demonstrated better interrater reliability between neuropathologists.^35^

Sixty‐three decedents without B score and/or neuritic plaque C score assessments were excluded. They had died less recently (2001 [11.7] vs 2006 [8.2], *p* = 0.002 with independent, two‐sided *t* test at *α* = 0.05) and were younger at death (76.5 [11.5] vs 79.3 [8.9], *p* = 0.02) compared to those included. For a sensitivity analysis, we included females (*n* = 556; increasing the overall sample to *n* = 1153) and arrived at conclusions consistent with the primary results. Similar to limitations in previous studies,^37^ AD neuropathologic change (following NIA‐AA criteria) was not evaluated since Thal phase measurement of amyloid plaques (A score) was only available for a relatively small sample donating 2014 and after (*n* = 117).

**RESEARCH IN CONTEXT**

1. **Systematic review**: Those with prior military service have greater exposure to AD risk factors, but whether AD neuropathological burden is higher than the general population is unknown.
2. **Interpretation**: Public data tracing revealed that 60% of the male sample had a history of military service. Modeled analyses suggest those with prior military service have greater odds of AD‐related neuropathology at autopsy including amyloid plaques.
3. **Future directions**: In the era of anti‐amyloid therapies, greater pushes by Alzheimer's Disease Research Centers (ADRCs) and the US Department of Veterans Affairs (VA) to increase veteran representation in AD research is critical to understand (a) amyloid prevalence in the veteran population relative to the general population and (b) the role that the military exposome has in shaping AD risks, including amyloid positivity. Only then will providers be equipped with the best strategies to identify and treat veterans at greatest risk for AD.

### 2.4 Analysis

Descriptive statistics are provided for the sample. Generalized ordered logistic regression^38^ was used to model the ordinal B score and C score separately, with site‐level clustered standard errors adjusting for covariates regularly available across the 33‐year data time frame: age and year of death ≥2005 (ie, year that the National Alzheimer's Coordinating Center introduced standardized data collection and reporting activities using the Uniform Data Set at ADRCs). Sensitivity analysis was conducted that included both male and female decedents adjusted for age, sex, and year of death. The method involved running a series of logistic regressions comparing the odds at increasing levels of the outcome. For C score analysis, there were two different comparisons conducted given the existing data: (sparse, moderate, or frequent) versus (no plaques) and (moderate or frequent) versus (no plaque or sparse). For B score analysis, there were three comparisons: (B score 1, 2, 3) versus (B score 0), (B score 2, 3) versus (B score 0, 1), and (B score 3) versus (B score 0, 1, 2). The Brant test was used to identify individual variables that violated the proportional odds assumption across logistic regression model comparisons.^39^ When violated, this means that a variable's association is not the same across comparisons, and separate odds ratios are provided for each logistic regression. When the assumption is met, the odds ratio is the same across all comparisons. Data were analyzed using Stata/SE version 17.0 (StataCorp LLC).

## 3 RESULTS

**FIGURE 1:**
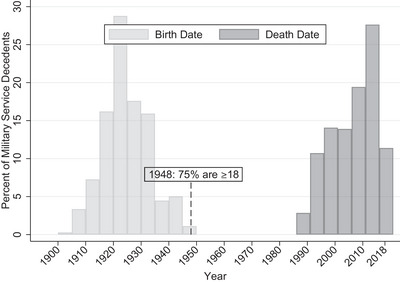
Birth and death year for decedent sample with military service.

Public record tracing determined that 60% of male decedents had a history of military service (Table 1). The majority of the male sample with military service had a median year of birth of 1923 (IQR = 1919 to 1930) and median year of death of 2007 (IQR = 1998 to 2012) (Figure 1). Therefore, 75% of those with a military history were 18 years of age by 1948.

**TABLE 1:** Characteristics of male decedent brain bank sample.

Characteristic	Overall sample (*n* = 597)	No military service (*n* = 239)	With military service (*n* = 358)
Mean age – year ± SD	79.3 ± 8.9	77.3 ± 9.9	80.7 ± 7.8
Age group – no. (%)			
≦69 years	89 (15)	58 (24)	31 (9)
70–79 years	198 (33)	77 (32)	121 (34)
80–89 years	241 (40)	75 (31)	166 (46)
≧90 yr	69 (12)	29 (12)	40 (11)
Year of death – no.(%)^a^			
1986 to 2004	230 (39)	83 (35)	147 (41)
2005 to 2018	367 (61)	156 (65)	211 (59)
B score			
B0: No neurofibrillary tangles	27 (5)	13 (5)	14 (4)
B1: Braak stage I/II	130 (22)	53 (22)	77 (22)
B2: Braak stage III/IV	106 (18)	41 (17)	65 (18)
B3: Braak stage V/VI	334 (56)	132 (55)	202 (56)
C Score			
C0: No plaque	94 (16)	43 (18)	51 (14)
C1: Sparse	33 (6)	14 (6)	19 (5)
C2/C3: Moderate/frequent	470 (79)	182 (76)	288 (80)

^a^ Categorized before and after 2005 because that is the year that the National Alzheimer's Coordinating Center began implementing the uniform data collection across ADRC brain banks.

Following adjustments for age and death year, prior military service was associated with a 26% increased adjusted odds of having a higher neuritic amyloid plaque C score (Table 2; OR = 1.26, 95% confidence interval [CI]: 1.06 to 1.49). This 26% adjusted odds increase applies to increasing levels of the C score [when comparing (sparse, moderate, or frequent) versus (no plaques) and when comparing (moderate or frequent) versus (no plaques or sparse)].

**TABLE 2:** Adjusted odds of Alzheimer's neuropathology features associated with military service history.

	Neuritic amyloid plaque C score^a^		Neurofibrillary tangle B score^b^		
	Adjusted OR (95% CI)		Adjusted OR (95% CI)		
Characteristic	C score Sparse, moderate, frequent versus no plaque	C score Moderate, frequent versus no plaque, sparse	B score 1, 2, 3 versus 0	B score 2, 3 versus 0, 1	B score 3 versus 0, 1, 2
Military service history					
No	1 [Reference]	1 [Reference]	1 [Reference]	1 [Reference]	1 [Reference]
Yes	1.26 (1.06 to 1.49)	1.26 (1.06 to 1.49)	1.10 (1.08 to 1.12)	1.10 (1.08 to 1.12)	1.10 (1.08 to 1.12)
Age grouping					
<70	1 [Reference]	1 [Reference]	1 [Reference]	1 [Reference]	1 [Reference]
70 to 79	1.51 (1.30 to 1.76)	1.51 (1.30 to 1.76)	2.37 (1.83 to 3.05)	1.41 (1.34 to 1.49)	0.92 (0.65 to 1.32)
80 to 89	1.36 (0.94 to 1.95)	1.36 (0.94 to 1.95)	4.72 (2.29 to 9.73)	1.64 (1.45 to 1.86)	0.79 (0.36 to 1.74)
≥90	0.97 (0.77 to 1.22)	0.97 (0.77 to 1.22)	8.06 (3.75 to 17.31)	1.16 (1.02 to 1.33)	0.41 (0.31 to 0.54)
Year of death ≥2005^c^	1.16 (0.54 to 2.50)	1.36 (0.72 to 2.59)	1.26 (1.11 to 1.43)	1.26 (1.11 to 1.43)	1.26 (1.11 to 1.43)

*Notes*: Cell values denote OR and 95% CIs produced from generalized ordered logistic regression models. Adjusted model includes all variables listed in table (age and year of death). For each analysis, ORs are the same in each logistic regression if the proportional odds assumption was met, whereas ORs are different across logistic regressions when the proportional odds assumption was violated.

Abbreviations: CI, confidence intervals; OR, odds ratio.

^a^ For C‐score analyses, the proportional odds assumption was met for military service and all age groups (ORs were the same across C‐score comparisons). It was violated for year of death ≥2005 (OR varied depending on C‐score comparison).

^b^ For B‐score analysis, the proportional odds assumption was met for military service and year of death. It was violated for all age groups (OR varied depending on B‐score comparison).

^c^ Categorized before and after 2005 because that is the year that the National Alzheimer's Coordinating Center began implementing the uniform data collection and reporting across ADRCs.

In addition, having a military service history is strongly associated with *post mortem* AD tau pathology. Specifically, there is a 10% greater adjusted odds of a higher neurofibrillary tangle B score compared to those without a history of military service (Table 2; OR = 1.10, 95% CI: 1.08 to 1.12). This 10% odds increase applies to each increasing level of the B score [at B score (1, 2, 3) versus (0), B score (2, 3) versus (0, 1), and B score (3) versus (0, 1, 2)].

In sensitivity analyses that expand the sample to include male and female decedents, public data tracing found 32% had prior military service (15 female, 358 male decedents with military service history). In both C and B score analyses, adjusted odds ratios related to military service history were similar to the male‐only sample estimations (C score OR = 1.24, 95% CI: 1.13 to 1.37; B score OR = 1.12, 95% CI: 1.11 to 1.13).

## 4 DISCUSSION

In this new era of anti‐amyloid medications, efforts to categorize amyloid positivity in the VA population are increasingly relevant. To the best of our knowledge, this study is the first to link a history of military service to AD neuropathology, providing important evidence for the need for amyloid screening within the veteran population. Yet many investments are needed to ready the Veterans Health Administration infrastructure to support such screening. From a research perspective, understanding the ways in which the military exposome affects AD biological processes is essential. Such research raises awareness of the need for actionable clinical policies, whereby findings inform interventions (such as anti‐amyloid therapies) targeted to maximize health.

There are several study limitations. ADRC cohorts are recruitment‐based, so selection bias in brain donation is likely.^40^ Prevalence estimation of AD neuropathologic burden and multilevel mechanistic discoveries will require coordinated involvement between ADRCs and VA for larger, more generalizable samples in veteran and non‐veteran populations. Our findings emphasize the urgent need to fill this research gap. This includes detailed sex differences. Recent ADRC and VA initiatives to increase veteran representation in AD research underscore this pressing need, and diverse veteran recruitment should continue to expand. Given the limited information characterized for the sample, we were unable to rigorously identify mediating and moderating factors that may explain why individuals with military service are at greater risk of having amyloid and tau neuropathology (including the interplay between environmental and genetic risk factors such as apolipoprotein E [APOE] status). Military service exposures have been associated with posited AD mechanisms, including chronic stress and post‐traumatic stress disorder (PTSD), traumatic brain injury, and cardiovascular risk factors.^10^, ^11^, ^12^, ^13^, ^14^, ^15^, ^16^, ^17^, ^18^, ^19^, ^20^, ^21^ Efforts will also require additional data linkages and strong data sharing agreements with the Veteran Benefits Administration and Department of Defense to evaluate the multiple, overlapping pathways impacting the molecular and cellular processes involved in AD. This includes evaluating dose‐response relationships, identifying subgroups at heightened risk, and exploring cohort effects across time. Such agreements will also allow for a deeper characterization of the military exposome and inclusions of pre‐ and post‐service risk factors along pathways.

Results also suggest public data tracing has the potential to change the way the military exposome is studied. Additional investments are needed to characterize other ADRD and other AD‐focused biobank cohorts as well as to evaluate whether the method can be used to extract more detailed service exposures. Validation assessments are also needed to establish the method's criterion validity (eg, among those who received VA benefits) and evaluate issues of misclassification.

With this study suggesting veterans have an increased risk of amyloid and neurofibrillary tangle burden, results emphasize that targeted AD therapies in the veteran population are urgently needed.

## CONFLICT OF INTEREST STATEMENT

The authors declare no conflicts of interest. Author disclosures are available in the supporting information.

## Supporting information



Supporting Information
